# Diabetic Enteropathy: From Molecule to Mechanism-Based Treatment

**DOI:** 10.1155/2018/3827301

**Published:** 2018-09-16

**Authors:** Theresa Meldgaard, Søren Schou Olesen, Adam D. Farmer, Klaus Krogh, Anne Astrid Wendel, Birgitte Brock, Asbjørn Mohr Drewes, Christina Brock

**Affiliations:** ^1^Mech-Sense, Department of Clinical Medicine, Aalborg University, Department of Gastroenterology & Hepatology, Aalborg University Hospital, Mølleparkvej 4, 9000 Aalborg, Denmark; ^2^Centre for Digestive Diseases, Blizard Institute of Cell & Molecular Science, Wingate Institute of Neurogastroenterology, Barts and the London School of Medicine & Dentistry, Queen Mary University of London, London, 4 Newark Street, London E1 2AT, UK; ^3^Department of Gastroenterology, University Hospitals of North Midlands, Stoke-on-Trent, Staffordshire ST4 6QJ, UK; ^4^Department of Hepatology and Gastroenterology, Aarhus University Hospital, Palle Juul Jensens Boulevard, 8200 Aarhus N, Denmark; ^5^Steno Diabetes Center Copenhagen, The Capital Region of Denmark, Niels Steensens Vej 2-4, Building: NSK, 2820 Gentofte, Denmark

## Abstract

The incidence of the micro- and macrovascular complications of diabetes is rising, mirroring the increase in the worldwide prevalence. Arguably, the most common microvascular complication is neuropathy, leading to deleterious changes in both the structure and function of neurons. Amongst the various neuropathies with the highest symptom burden are those associated with alterations in the enteric nervous system, referred to as diabetic enteropathy. The primary aim of this review is to provide a contemporaneous summary of pathophysiology of diabetic enteropathy thereby allowing a “molecule to mechanism” approach to treatment, which will include 4 distinct aspects. Firstly, the aim is to provide an overview of the diabetes-induced structural remodelling, biochemical dysfunction, immune-mediated alterations, and inflammatory properties of the enteric nervous system and associated structures. Secondly, the aim is to provide a synopsis of the clinical relevance of diabetic enteropathy. Thirdly, the aim is to discuss the various patient-reported outcome measures and the objective modalities for evaluating dysmotility, and finally, the aim is to outline the clinical management and different treatment options that are available. Given the burden of disease that diabetic enteropathy causes, earlier recognition is needed allowing prompt investigation and intervention, which may lead to improvements in quality of life for sufferers.

## 1. Introduction

The increasing incidence of both type 1 and type 2 diabetes elevates the complications of diabetes as one of the most important current public health issues [1], which causes negative impact on the individual quality of life and increased socioeconomic expenditure. Amongst the diabetic complications with the highest symptom burden, yet frequently underrecognised and suboptimally treated, are those associated with alterations in the enteric nervous system (ENS), hereinafter referred to as diabetic enteropathy. This review will focus on a “molecule to mechanism” approach of diabetic enteropathy and mechanism-based treatments.

## 2. The Enteric Nervous System

This review will provide a detailed summary of the remodelled and dysfunctional wall of the gastrointestinal (GI) tract and the resulting pathological complications. These include (1) reduced number of intrinsic enteric neurons, (2) structural neuronal changes, (3) intraneuronal biochemical changes, (4) diminished secretion of neurotransmitters, (5) altered immunomodulatory function of the enteric glial cells, (6) neuroinflammation, and (7) altered gut-brain communication through spinal afferents and vagal terminals. These concomitant changes cause altered GI motility and secretory functions and explain—at least partly—the development and maintenance of nausea/vomiting, bloating, early satiety, diarrhoea, constipation, and abdominal pain.

**Figure 1:**
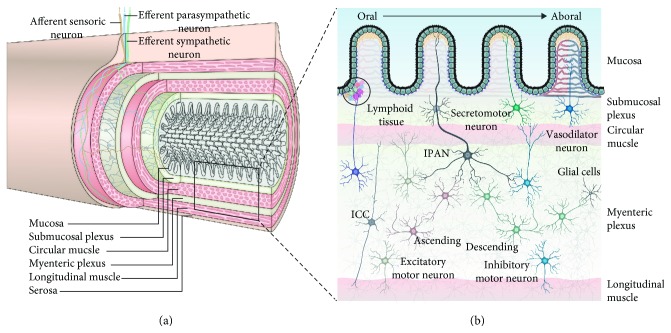
The enteric nervous system. (a) Cross-sectional view. The enteric nervous system (ENS) is embedded in the wall of the GI tract. The neurons are localized in the myenteric and submucosal plexi and are connected by interneurons (depicted in grey). Extrinsic efferent innervation via autonomic sympathetic (green) and parasympathetic (blue) pathways contributes to the regulation and coordination of GI function. Extrinsic afferent sensory nerves (orange) following either vagal or spinal routes provide the central nervous system with information about GI homeostasis. (b) Longitudinal view illustrating a selection of neuronal subtypes. Secretomotor and vasodilator neurons regulate fluid and molecular exchange between gut lumen, tissue, and vasculature. Peristaltic movements (oral contraction and aboral relaxation of intestinal smooth muscle) are facilitated by intrinsic primary afferent neurons (IPANs) activating ascending and descending interneurons, which then activate upstream excitatory and downstream inhibitory motor neurons, respectively. IPANs may initially be activated, e.g., through mechanoreceptors or by acetylcholine secreted by enteric endocrine cells in the luminal epithelial cell layer upon luminal distension. In addition, ENS includes the innervation of gastroenteropancreatic endocrine cells (not shown) and gut-associated lymphoid tissue, responsible for hormone secretion and transmitter release. Although not equally represented, the juxtapositioned networks of enteric glial cells (EGCs) and interstitial cells of Cajal (ICCs) are present in all layers of the GI wall. Note that the thickness of the different tissue layers is not proportionally represented.

The ENS consists of a complex network of neurons and enteric glial cells (EGCs), which are embedded in the wall of the GI tract. The neurons are localized in the myenteric and submucosal plexi, which are connected by interneurons. The myenteric plexus is situated between the circular and longitudinal muscle layers and influences GI motility. The submucosal plexus is in close proximity to the muscularis mucosae, intrinsic vasculature, and the mucosa [2] (Figure 1(a)) and regulates the secretion of hormones and neurotransmitters. Furthermore, local sensory neurons called intrinsic primary afferent neurons (IPANs) regulate motility and maintain homeostasis. The ENS is supplemented with extrinsic efferent input from the central nervous system via autonomic (both sympathetic and parasympathetic) pathways which also contribute to the regulations and coordination of GI function [3]. Although the majority of enteric afferent axons are confined to the gut wall, a large amount of sensory neurons from the CNS following either vagal or spinal routes have receptive fields in different layers of the GI wall and monitor GI homeostasis [4]. Approximately 80–85% of the nerve fibres in the vagus nerve are afferent and project viscerotopically to the nucleus of the solitary tract [5].

Neurons of the ENS can be categorised according to their connectivity and function (Figure 1(b)). The interstitial cells of Cajal (ICCs), whilst not strictly neuronal, generate and convey electrical impulses to smooth muscle cells facilitating the slow wave peristaltic movement of the stomach and intestines and are referred to as “pacemaker” cells [6].

In summary, the ENS comprises of three panenteric juxtapositioned networks, namely, neurons, EGCs, and ICCs. The detailed role of EGCs is discussed below; however, both enteric neurons and EGCs are particularly vulnerable to hyperglycaemia.

## 3. Diabetic Enteropathy

Diabetes significantly alters the microenvironment within the ENS due to the effect of, amongst other hyperglycaemia, oxidative stress, neuroinflammation, reduced levels of nerve growth factors, and structural vascular changes [7–9]. In addition, other aspects such as increased levels of fatty acids, miRNA, endothelia dysfunction or altered enteric microbiota also have been proposed to exert an influence [10] although these are outside the scope of this review.

### 3.1. Diabetes and Intracellular Biochemical Changes

Neurons have continuously high glucose demand. They cannot allow glycolytic and anaerobic episodes and are further provided with a physiology that fails to regulate episodic glucose uptake under the influence of insulin. Therefore, the neuronal glucose uptake and utilization is highly dependent on the extracellular glucose concentration and facilitated diffusion mediated by primarily glucose transporter 3 (GLUT3); however, other forms including GLUT1, GLUT4, and GLUT8 are also present [11]. Hyperglycaemia in diabetes causes up to fourfold increases in glucose levels, and if this is persistent or repetitive, then intracellular glucose metabolism leads to neuronal damage often referred to as glucose neurotoxicity [12]. These mechanisms are primarily described in the peripheral and central nervous systems, but the same mechanisms are present in the enteric nervous system.

**Figure 2:**
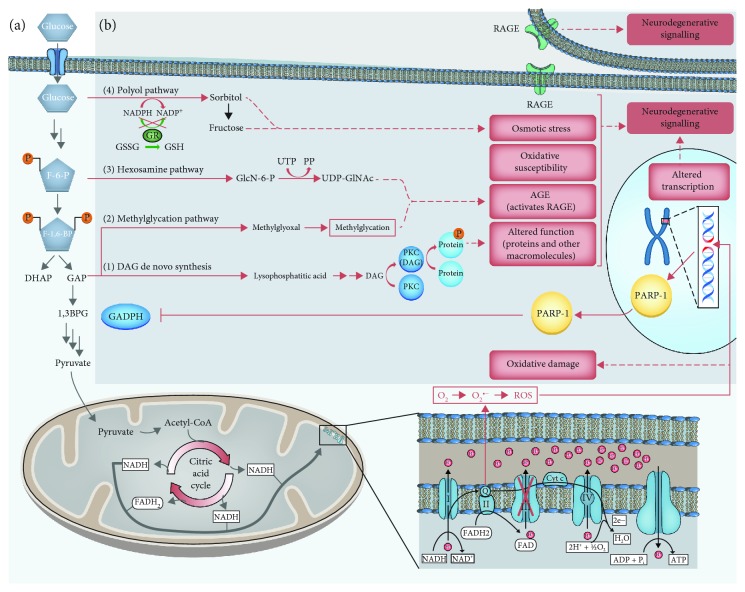
Hyperglycaemia induced intracellular biochemical changes in neurons. (a) Generation of ROS. (b) Consequences of ROS generation. See text and Table 1 for explanation. Abbreviations: 1,3BPG: 1,3-bisphosphoglyceric acid; Acetyl-CoA: acetyl coenzyme A; ADP: adenosine diphosphate; AGE: advanced glycation end products; ATP: adenosine triphosphate; DAG: diacylglycerol; DHAP; dihydroxyacetone phosphate; e^−^: electron; F-1,6-BP: fructose-1,6-bisphosphate; F-6-P: fructose-6-phosphate; FAD: flavin adenine dinucleotide (oxidised); FADH_2_: flavin adenine dinucleotide (reduced); GAP: glyceraldehyde 3-phosphate; GAPH: glyceraldehyde 3-phosphate dehydrogenase; GlcN-6-P: glucosamine 6-phosphate; GR: glutathione reductase; GSH: glutathione; GSSG: glutathione disulphide; H^+^: proton; NAD^+^: nicotinamide adenine dinucleotide (oxidised); NADH: nicotinamide adenine dinucleotide (reduced); NADP^+^: nicotinamide adenine dinucleotide phosphate (oxidised); NADPH: nicotinamide adenine dinucleotide phosphate (reduced); O_2_: oxygen: O_2_^•−^: superoxide; P: phosphor group; PARP-1: poly(ADP-ribose) polymerase 1; PKC: protein kinase C; PP: diphosphate; RAGE: receptor for advanced glycation end products; ROS: reactive oxygen species; UDP-GlcNAc: uridine diphosphate N-acetylglucosamine; UTP: uracil triphosphate.

The increased glucose flow through the glycolytic pathway leads to increased levels of pyruvate, which is oxidised in the citric acid cycle. This initiates a continuous elevated flux of electron donors (NADH and FADH_2_) into the electron transport chain. Subsequently, this leads to an increased voltage gradient across the inner mitochondrial membrane, caused by the efflux of protons from the mitochondrial matrix into the intermembrane lumen by complexes I, III, and IV [13]. At a critical membrane potential threshold, the electron transfer of complex III stalls [13], causing coenzyme Q to donate electrons to molecular oxygen, which generates superoxide, denoted O_2_^•−^ (Figure 2(a)). Superoxide is a reactive oxygen species and drives the delirious effects of increased intracellular glucose concentrations, which ultimately leads to oxidative stress and tissue damage. Interestingly, different populations of neurons have differing degrees of susceptibility to glucose-initiated oxidative stress, which results in pleomorphic neurological sequelae.

Animal models have shown that during hyperglycaemic episodes, the extrinsic sympathetic supply, via coeliac and superior mesenteric ganglia to the ENS, is more sensitive than those deriving from the superior cervical ganglion [14]. Such susceptibility has a number of consequences (Figure 2(b)). Reactive oxygen species cause DNA double-strand breaks, which in return activates DNA repair mechanisms, including the enzyme PARP-1. Activated PARP-1 inhibits the key glycolytic enzyme glyceraldehyde 3-phosphate dehydrogenase. This causes the accumulation of upstream glycolytic intermediates [15], which then are diverted into alternative and ultimately pathogenic pathways (Table 1). Glycation of various intra- and extracellular proteins and lipids ultimately results in the formation of advanced glycation end products. Advanced glycation end products activate receptor for advanced glycation end products on the affected and surrounding cells, including myeloid cells, initiating inflammatory and hence neurodegenerative signalling [16]. Depletion of intracellular NADPH renders the neuron susceptible to oxidative damage due to lacking regeneration of the antioxidant glutathione, thereby resulting in a “vicious” cycle.

Over time, the biochemical alterations coalesce with neuronal structural changes and endothelial dysfunction to drive the pathological development of diabetic neuropathy.

### 3.2. Diabetes and Enteric Neuroinflammation and Oxidative Stress

Inflammation and oxidative stress are two synergistic conditions, which have a significant negative impact on the function of the ENS. *In vitro* studies of human EGCs have demonstrated that inflammation induces proinflammatory pathways leading to alterations in functional signalling pathways linked to GI motility such as mechanical-evoked Ca^2+^ and purinergic signalling [17], indicating that GI dysfunction may indeed be related to inflammation. Whilst such findings have not been comprehensively studied in clinical populations of diabetes, animal models have shown associations between increased oxidative stress and gastroparesis, which could be prevented by treatment with antioxidants [18]. Similar observations have been made in the jejunal tissue of rats with diabetes, where loss of both neurons and EGC was significantly reduced after 120 days of supplement with the antioxidant quercetin [19]. However, trials of antioxidants in adults with diabetes have, to our knowledge, not yet been successful in preventing or improving GI symptoms.

### 3.3. Diabetes and Structural Neuronal Changes

In animal models of streptozotocin-induced diabetes (streptozotocin is a drug that has preferential toxicity against pancreatic *β* cells), there is marked degeneration, coupled with a reduction in the density, of neurons in the myenteric plexus [20–24]. In adults with diabetes, there is a reduction in the quantity of colonic ENS, assessed as a total ganglion area by immunohistochemical staining, in comparison to healthy controls [25]. Notably, autonomic neurons, including the ENS, are particularly vulnerable to hyperglycaemia [26]. It has been suggested that diabetes preferentially affects large fibre neurons in the dorsal root ganglion and inhibitory neurons in the gut wall. In particular, selective loss of nitric oxide synthase and neuropeptide Y-expressing inhibitory neurons has been shown in human diabetic colon [25]. However, since the overall motility, coordination, and GI homeostasis are affected in diabetes, it is plausible that IPANs are also vulnerable to chronic hyperglycaemia. Moreover, degeneration and/or loss of ICCs throughout the GI tract has been reported in both animal models and in patients [18, 27], causing reduced frequency of spontaneous muscular contractions. Finally, smooth muscle myopathy [28] and angiopathy [8] are considered a contributing factor in the development of diabetic enteropathy. Taken together, diabetes induces marked structural remodelling of the wall of the GI tract and its neuronal support leading to altered function of the GI tract.

### 3.4. Diabetes and Immunomodulatory Involvement of Enteric Glial Cells

Besides providing neurotrophic support, EGCs mediate interactions between enteric neurons and other cell types. Through a number of processes, they communicate with immune effector cells, enteroendocrine cells, epithelial cells, and blood vessels, forming a “circuit” that specialises in the control and integration of bidirectional signals from neurons to other cells [29]. Although EGCs exert immunosuppressive and anti-inflammatory effects protecting the ENS against intraluminal foreign antigens, the physiological role of each subtype is still incompletely understood [30]. For example, it has been shown that in diabetes, loss of EGCs throughout the GI tract influences GI function directly. Associated to this, a decreased secretion of neurosupportive factors has been observed [31]. For instance, glial cell line-derived neurotrophic factor mediates differentiation and migration of enteric neurons as well as survival and protection against the adverse effects of hyperglycaemia through the activation of the neuron-specific Ret tyrosine kinase receptor and coupled PI3K and MAPK pathways in neurons [31, 32]. Taken together, loss of EGC leads to neuronal neglect and apoptosis in the diabetic ENS.

## 4. Clinical Aspects

As previously described, diabetes results in the loss of neurons causing dysmotility and altered secretion within the entire GI tract and therefore diabetic enteropathy should be considered as a panenteric disorder. For example, oesophageal motor disorders in persons with diabetes has a reported prevalence of up to 63%, which is greater than that of gastroparesis (13%) [33]. A common secondary complication to oesophageal motor disorders is gastroesophageal reflux disease. However, the most thoroughly described GI complication is gastroparesis, defined as delayed gastric emptying in the absence of mechanical gastric outlet obstruction. It is estimated that 5% of adults with type 1 diabetes and 1% of adults with type 2 diabetes develop gastroparesis after 10 years of disease duration [34]. The cardinal symptoms of gastroparesis are early satiety, postprandial fullness, bloating, nausea, pain, vomiting, and weight loss [35]. However, there is considerable interindividual variability in symptoms between patients, with symptom severity being related to the duration of diabetes [35] and poor glycaemic control [35, 36]. From a mechanistic point of view, acute hyperglycaemia reduces the rate of gastric emptying and increases the sense of fullness during gastric distension directly [37, 38]. Changes in gastric emptying lead to unpredictable delivery of nutrition (and thus glucose) and oral pharmacotherapeutic agents into the small bowel [39]. However, it is not known whether chronic poor glycaemic control is the cause or the consequence of gastroparesis, but in reality, it is likely that these factors interact with one another. Notwithstanding the significant symptom burden, gastroparesis is also associated with significant healthcare expenditure. Notably, clinical examinations and hospitalizations due to gastroparesis are increasing as well as the length of stay [40].

The correlation between visceral neuropathy and GI symptoms remains incompletely understood [41]. Lower GI symptoms in adults with diabetes are common, with a twofold increase in the risk of experiencing constipation, diarrhoea, and faecal incontinence [34]. The prevalence increases with poor glycaemic control, and both hard stools and faecal incontinence are reported four times more often in patients with poor diabetes regulation than in those who are with well-regulated diabetes [34]. Several studies have compared the prevalence of GI symptoms amongst adults with diabetes types 1 and 2, but no consistent differences have been found [35].

## 5. Patient-Reported Outcome Measures

Although patients' experience and perceptions are central to the clinical evaluation, patient-reported outcome measures/questionnaires are helpful in both research and the longitudinal monitoring of response to interventions. The most commonly used are Patient Assessment of GI Disorders-Symptom Severity Index (PAGI-SYM), Gastroparesis Cardinal Symptom Index (GCSI), Gastrointestinal Symptom Rating Scale (GSRS), the Patient Assessment of Constipation Symptoms (PAC-SYM), and Patient Assessment of Constipation-Quality of Life (PAC-QoL).

### 5.1. PAGI-SYM

PAGI-SYM assesses the severity of common upper GI symptoms. This validated instrument contains 20 items and assesses six subscales: heartburn/regurgitation, postprandial fullness/early satiety, nausea/vomiting, bloating, upper abdominal pain, and lower abdominal pain. This questionnaire allows monitoring of outcomes in clinical practice and trials and is a reliable instrument in subjects with gastroesophageal reflux disease, dyspepsia, or gastroparesis [42].

### 5.2. GCSI

GCSI consists of three subscales measuring nausea/vomiting, postprandial fullness/early satiety, and bloating derived from the PAGI-SYM. It is based on a 2-week recall, has been validated, and is reliable in assessing symptom severity related to gastroparesis [43]. Conflicting results have been reported concerning the association between upper GI symptoms, as measured by GCSI, in diabetes and objective measures of gastroparesis, e.g., scintigraphic measures of gastric emptying [44–46]. However, the recent study confirmed that the severity of early satiety and postprandial fullness are associated with prolonged gastric emptying [47]. To evaluate the responsiveness to treatment of gastroparesis in clinical trials, the Gastroparesis Cardinal Symptom Index-Daily Diary (GCSIDD) was developed and validated.

### 5.3. GSRS

GSRS evaluates a wide range of GI symptoms. The questionnaire contains 15 items which are combined into five symptom clusters, namely, reflux, abdominal pain, indigestion, diarrhoea, and constipation [48]. However, the link between GSRS score and objective measures needs a further study. In a clinical setting, the GSRS gives a broader perspective on patients' panenteric GI symptoms in general, in comparison to the GCSI score, which focus on gastroparesis.

### 5.4. PAC-SYM and PAC-QoL

PAC-SYM and PAC-QoL were developed to evaluate symptom severity and quality of life in patients with constipation. The PAC-SYM is composed of 12 items with three subscales: abdominal symptoms, stool symptoms, and rectal symptoms. It is valid and reliable in the assessment of the presence and severity of constipation symptoms in adults over time as well as the ability to distinguish between responders and nonresponders to treatment [49]. A modified version (M-PAC-SYM) excluding item 7 (rectal bleeding/tearing) has been developed for patients with chronic constipation [49] and may be more relevant for the evaluation of functional constipation in diabetes. The PAC-QoL is a validated and consistent questionnaire [50]. It includes 28 items forming four subscales (worries and concerns, physical discomfort, psychosocial discomfort, and satisfaction) and an overall scale, and thus, it is comprehensive in assessing the burden of constipation on patients' well-being and everyday functioning. In diabetes, there is an existing knowledge gap on the presence of prolonged colonic transit and constipation and the potential implication on the experienced burden. Moreover, bioavailability of nutrition and on pharmacotherapeutic and glycaemic control warrants further investigation [51].

## 6. Modalities for Assessing Motility

A number of modalities exist for objectively evaluating diabetic enteropathy. For detailed assessment of GI disorders, objective investigations are a necessary supplement to subjective assessments. Although few has been strictly validated, the most common tests are reviewed here.

### 6.1. Scintigraphy

Scintigraphic evaluation of gastric emptying is considered to be the gold standard for investigating gastroparesis. This is a quantitative method in which the patient ingests a 99-technicium-radiolabelled standardized meal following which gastric emptying is measured. Although widely available, differences in the delivery of the test and its interpretation have limited the interpretation of results between centres although significant efforts, e.g., from the American Neurogastroenterology and Motility Society and Society of Nuclear Medicine, have aided in the standardization of the performance and interpretation of gastric emptying [52]. Associations between scintigraphic results of gastric emptying and clinical experienced symptoms are poor but have been shown between gastric emptying and fullness/early satiety and nausea/vomiting [53]. Scintigraphy can also be used to measure small bowel transit time and colonic transit time although this requires prolonged sequential scanning and is largely limited to a number of tertiary centres.

### 6.2. Breath Testing

Nonradioactive ^13^C isotope bound to a digestible substance, most commonly octanoic acid, can be used as a proxy of gastric emptying. ^13^C octanoic acid is mixed with a solid meal and ingested, where it is absorbed from the proximal small intestine. Subsequently, it is metabolized in the liver to ^13^C-CO_2_ and can be measured in exhaled breath. Breath testing demonstrates good receiver operator characteristics (sensitivity of 89% and specificity of 80%) in comparison to scintigraphy [54]. In comparison to scintigraphy, ^13^C octanoic acid does not radiate and sampling can be undertaken in the waiting room. However, concomitant small bowel pathology, such as coeliac disease, can affect breath testing results.

### 6.3. Manometry

Diabetes affects the oesophageal motility, but studies show contradictive findings covering normal oesophageal motility, delayed oesophageal transit times, and reduced pressure of the lower oesophageal sphincter [55]. However, most of these studies have used conventional manometry catheters, which do not allow for continuous pressure monitoring throughout the oesophagus. In contrast, a newer study in type 2 diabetes used high-resolution oesophageal manometry and found that gastric emptying and oesophageal motility were not generally altered, which possibly suggests that the previous reported extent of gastrointestinal disorders in patients with diabetes may now be reduced due to improved standards of care [56] and better glycaemic control. Furthermore, high-resolution colonic manometry has been used to evaluate physiology and pathophysiology of constipation. Thus, the methodology has been suggested to evaluate the gastrocolonic response, which is potentially mediated by extrinsic neural pathways, and therefore, an absent response could indicate neuropathy in the extrinsic colonic efferents [57, 58].

### 6.4. Wireless Motility Capsule

This system comprises of an indigestible capsule that continuously measures pressure, temperature, and pH as it traverses the GI tract. Based on stereotypical changes in temperature and pH, segmental and panenteric transit times can be derived [59]. The test involves a standardized meal following which the patient ingests the capsule; data is transmitted wirelessly to a receiver unit worn by the patient until it is expelled. There are a set of robust normal values, and its use has been approved by the Food and Drug Administration [60]. In one study in patients experiencing GI symptoms, 65% had prolonged gastric emptying, 24% had prolonged small intestinal transit time, and 58% had prolonged colon transit time [61]. The findings mirrored another cohort of adults with diabetes and established sensorimotor neuropathy, where 44% had abnormal transit in one or more segments, independent of symptomatology [51]. Therefore, accumulating evidence supports that gastroparesis can coexist with prolonged transit in the small and large bowels as well as low contractility of the colon [61–63]. Beyond pure measurement of transit times, it has recently been proposed that the change in pH across the ileocaecal junction may represent a surrogate marker for caecal fermentation, which itself may influence colonic transit times [51]. Heightened bacterial fermentation in the caecum increases the quantity of short-chain fatty acids, which results in regional acidification. In the future, this may represent a potential therapeutic target in adults with diabetes. In contrast to breath testing, the WMC method provides valuable knowledge such as orocaecal transit [64]; it is, however, more expensive and limited to specialist centres.

### 6.5. Radiopaque Markers

Radiopaque markers (ROM) are capsules containing plastic beads or rings that are ingested by the patients following which a plain abdominal radiograph is undertaken. Although various protocols exist in terms of the number of capsules to be taken and the number of radiographs undertaken, it is a useful method to delineate whole gut transit time and by proxy colonic transit time as this is the major component of the former [65]. A gastric emptying test with ROM is a widely available screening method to detect delayed gastric emptying in adults with diabetes, where a positive result seems reliable. However, a normal ROM test does not exclude delayed gastric emptying, and if the clinical suspicion of gastroparesis remains, scintigraphy should be performed [53].

### 6.6. Emerging Techniques

Emerging techniques such as magnetic resonance imaging, the 3D transit system, and the video capsule endoscopy are being developed to assess transit times and motility [66]. Magnetic resonance imaging involves repeated T2-weighted images being recorded by using nonrigid image registration in regional areas of interest, and small and large bowel motor function can be elucidated [67]. The 3D transit system allows continuous tracking of an electromagnetic capsule ingested by a patient relative to an external plate worn on the abdomen. In addition to transit times, the system allows measurement of the speed, direction, and duration of motility [68]. The video capsule endoscopy is widely used clinically, for instance, to investigate occult gastrointestinal bleeding [69]. Developments in automated software analysis have allowed the systematic quantification of the motion and dynamics of the small bowel [70]. However, whilst these novel techniques offer distinct advantages over and above established methods, further work is needed to define normative values as well as the relation of findings to patient symptoms.

In summary, although most studies have focused on gastroparesis and GI symptoms in diabetes, newer studies show panenteric multisegmental prolongation of transit times prior to the development of clinical symptoms. Taken together, associations between clinical symptoms and sensory abnormalities of the GI tract show conflicting results, which mirrors the neuronal complexity of the ENS, spinal afferents, and central modulation. In the recent study, the evaluation of the brain-gut axis was investigated in adults with diabetes and GI symptoms. The authors provided evidence for the interaction between autonomic neuropathy and peripheral nervous degeneration, as well as changes in the brain processing [71, 72]. Therefore, clinical GI symptoms may not originate from the GI tract but can be developed and maintained through altered central processing. However, in the clinical setting, proactive encouragement of patients to modify lifestyle factors such as improved glycaemic control, daily water intake, dietary aspects, and physical exercise should be emphasized in order to minimise the symptoms of diabetes-induced gut dysmotility.

## 7. Clinical Management

Diabetic enteropathy has no known cure. The goals of treatment are therefore to slow the progression, relieve symptoms, manage complications, and restore function. The key to preventing or delaying neuropathy is primarily through tight glycaemic control. Such targeted management guided by age, disease duration, and overall health may even improve current symptoms. Dietary and lifestyle advice can give persons with diabetes the tools for better control. Glycaemic control may also improve by the usage of an insulin pump in persons with insulin-dependent diabetes, or sometimes, the preprandial insulin should be given after the meal or in reduced amount when gastroparesis is present. Recently, continuous glucose monitoring devices that allow for glucose readings in real time have become available. Use of continuous glucose monitoring is recommended by national and international medical organisations and expert clinician consensus both in combination with pump and in persons on multiple daily insulin injections [73–76]. Both insulin pump and continuous glucose monitoring reduce the number of hyper- and hypoglycaemic events and thus are believed to be neuroprotective. Beyond optimizing hyperglycaemic control, no available treatments address the underlying polyneuropathy.

The treatment of GI symptoms deriving from diabetic enteropathy is challenging due to the multiple underlying mechanisms. An overview of some of the most frequently applied treatment possibilities is reviewed here.

### 7.1. Gastroparesis

#### 7.1.1. Nonpharmacological Management

Initial treatment of gastroparesis is based on dietary consulting and improvement of glycaemic control. To enhance emptying of the stomach, low soluble fibre, low fat, and small volume meals are recommended with protein supplementation as needed [77]. If standard dietary modifications are insufficient, small particle size diet as well as liquid and homogenized nutritional supplementations may be initiated with the reservation of postpyloric enteral tube feeding for the most severe cases [78]. Parenteral nutrition should be restricted to cases where all other nutritional treatment modalities have failed.

#### 7.1.2. Prokinetics

Prokinetics (Table 2) have been widely studied in the context of diabetic gastroparesis and generally shown effect in most studies [77, 79, 80]. However, it must be underlined that there is no absolute association between symptom improvement and changes in gastric motility after treatment with prokinetics [81] and most prokinetic drugs are limited to short-term use due to the risk of irreversible tardive dyskinesia (D_2_-receptor antagonists) and currently subjected to black box warnings from the FDA and EMA.

Future molecular targets to accelerate GI motility are currently identified, and relamorelin, a synthetic ghrelin analog, has shown promising results, as it increases growth hormone levels and accelerates gastric emptying [82]. Relamorelin has proven to be superior to placebo for symptom relief in phase IIA studies for diabetic gastroparesis, even though vomiting frequency was not reduced. Until today, relamorelin has been well tolerated and is safe in humans without cardiac or neurologic adverse effects, yet it is not approved by the Food and Drug Administration.

#### 7.1.3. Tricyclic Antidepressants

In a retrospective study, low-dose nortriptyline, amitriptyline, and desipramine have shown to reduce symptoms in patients with diabetes, chronic vomiting, and inadequate response to prokinetics [83]. However, in a large multicentre randomized controlled trial in adults with idiopathic gastroparesis, the use of nortriptyline (up to 75 mg per day) compared with placebo for 15 weeks did not improve the overall symptom score [84]. Thus, more evidence is needed to make any conclusive recommendations for diabetic gastroparesis.

#### 7.1.4. Endoscopic Procedures

Most endoscopic procedures evaluated for gastroparesis have been directed towards the pylorus, to investigate whether pylorus spasms may contribute to symptoms and delayed gastric emptying. Intrapyloric injection of botulinum toxin may transiently improve gastric emptying in patients with gastroparesis (idiopathic and diabetic), but after 1 month, the benefit was not superior to placebo [85], and in patients with idiopathic gastroparesis, botox was not superior to placebo in improving either symptoms or the rate of gastric emptying [86]. Other endoscopic procedures include transpyloric stenting and endoscopic myomectomy of the pylorus, but at present, there are no sufficient data to support these procedures outside protocol settings [77].

#### 7.1.5. Surgical Procedures

Gastric electrical stimulation (GES) has been approved by the FDA as a Humanitarian Device Exemption in patients with refractory symptoms of diabetic or idiopathic gastroparesis [87]. It shows most promising results in patients with predominance of nausea and vomiting, where response rates up to 60% are shown in uncontrolled studies. These results should, however, be interpreted cautiously because a controlled trial failed to show any difference in symptom scores between the on and off phases in patients treated for refractory diabetic gastroparesis [88]. The trial did, however, show a significant reduction in vomiting episodes during all phases compared with the preimplantation period as well as favourable long-term clinical outcome [89].

Other surgical options include total or subtotal gastrectomy, which generally should be reserved as a last resort treatment in patients with severe treatment refractory symptoms after thorough evaluation in a multidisciplinary setting. Importantly, the surgical reports reporting favourable outcomes of these procedures have all been performed in uncontrolled settings, with relatively short follow-up. Taken together, more studies are needed to elucidate the underlying mechanisms and potentially identify the patients that may benefit from surgical intervention.

### 7.2. Abnormal Bowel Function

The treatment options available for bowel dysfunction in patients with diabetic enteropathy follow the recommendations used for other functional GI disorders.

#### 7.2.1. Diarrhoea

In the presence of diarrhoea, patients should be evaluated for secondary causes including infectious and inflammatory bowel diseases, coeliac disease, exocrine pancreatic insufficiency, and small bowel intestinal overgrowth [90]. Fibre supplementation might be helpful in some cases but can also worsen symptoms of gastroparesis. In most cases, loperamide is an effective and safe treatment for chronic diarrhoea, although not formally evaluated in the context of diabetic enteropathy [91].

#### 7.2.2. Constipation

Treatment of constipation is based on conventional laxatives that all have been shown to be relatively efficient and safe [92]. In treatment of refractory cases, a more detailed workup may be needed which ideally should include assessment of intestinal transit time, endoscopy, proctography, and anorectal-physiological evaluation. In the presence of slow-transit constipation, osmotic laxatives are preferred over fibre supplementation and bulking agents, because they stimulate the intestines to absorb excessive amounts of fluid from the body. Novel treatment options include prucalopride [93] that may also improve symptoms of gastroparesis, and linaclotide may be particularly helpful in patients with concomitant symptoms of irritable bowel syndrome [94]. Gastric symptoms in diabetes may be caused—at least in part—by vagal neuropathy, and therefore, there is a theoretical background to use a neuromodulation treatment option and this is the rationale for gastric pacing. Several studies have shown that the method may be effective to alleviate nausea and vomiting and is cost-effective [95]. However, most studies have been small and suffered from methodological problems (i.e., no sham arm), and recent guidelines have not recommended this modality outside protocol studies [96]. In idiopathic faecal incontinence, emerging areas such as neuromodulation, e.g., sacral nerve stimulation, have shown promising results in other GI functional diseases [97]. Within diabetes enteropathy, data is sparse and currently, no randomized sham-controlled studies exist, but such studies will undoubtedly contribute with knowledge in the upcoming years.

### 7.3. Abdominal Pain

Treatment of abdominal pain secondary to diabetic enteropathy is complex and involves a multidisciplinary approach including diabetologist, gastroenterologist, pain specialist, and psychologists. An active screening for psychiatric comorbidity, including anxiety and depression, should be done, and treatment initiated if present. There is a paucity of studies investigating pharmacological therapies for pain associated with diabetic enteropathy. However, as the pain may be of neuropathic origin, drugs, which have been evaluated for this indication in other diseases, may be helpful. These include antidepressants (tricyclic antidepressants, selective serotonin reuptake inhibitors, and selective serotonin-noradrenaline reuptake inhibitors) as well as the gabapentoids (gabapentin and pregabalin) that can be used in combination depending on the clinical situation [98]. However, in many patients, the pain is secondary to transit problems, bacterial overgrowth, and constipation and shall be treated accordingly. Side effects to medications can also give abdominal pain, and if patients are treated with opioids on other indications, these may give bowel dysfunction and abdominal pain [99].

## 8. Concluding Remarks

Symptoms from the GI tract, including dysmotility and abdominal pain, are frequent in diabetes. Traditionally, diabetes-induced gastrointestinal complications are focusing solely on gastroparesis and symptoms of the upper gastrointestinal tract. However, accumulating evidence supports the presence of structural and functional alterations in the ENS of the entire GI tract and the interconnections with enteric glial cells and interstitial cells of Cajal. This explains the biochemical, immune-mediated, and inflammatory pathophysiological mechanisms, which coalesce in the development and maintenance of cell death, altered secretion of neurotransmitters, dysmotility, and concomitant symptoms in the entire length of the GI tract. Taken together, increased recognition of diabetic enteropathy may allow earlier diagnosis and intervention. This gives rise to hope for the recognition of diabetic enteropathy at an earlier time and specific diagnoses in the future. Finally, more targeted nonpharmacological and pharmacological treatments and interventions can be individually tailored based on pathophysiological findings, in order to improve patient outcomes.

## Figures and Tables

**Table 1:** Accumulation of upstream glycolytic intermediates and their consequences.

Glycolytic intermediate	Alternative pathway	Consequence
Glyceraldehyde-3-phosphate	(1) De novo synthesis of DAG	DAG activates protein kinase C resulting in altered intracellular phosphorylation levels
	(2) Glycosylation pathways	Glycation of various intra- and extracellular proteins and lipids^∗^
Fructose-6-phosphate	(3) Hexosamine pathway	
Glucose	(4) Polyol pathway	Leads to depletion of intracellular NADPH^∗∗^

^∗^ leads to formation of advanced glycation end products (AGE). ^∗∗^ renders the neuron susceptible to oxidation.

**Table 2:** Prokinetic for treatment of diabetic gastroparesis.

Drug	Mode of action	Recommended daily dose (formulation)	Comment
Metoclopramide	5-HT_4_ receptor agonist D_2_-receptor antagonist	10 mg TID (tablet)	Black box warnings for long-term use: (i) FDA < 3 months (ii) EMA ≤ 5 days
Domperidone	D_2_-receptor antagonist	10 mg TID (tablet) 30 mg BID (suppository)	Should be avoided in the presence of prolonged QT interval
Erythromycin	Motillin receptor agonist Cholinergic receptor agonist	250 mg TID (tablet)	Clinical efficacy often diminishes after 2–4 weeks due to tachyphylaxia Prokinetic action likely a drug class effect and other macrolides with less toxicity may be used (azithromycin, clarithromycin), but evidence from controlled trials is lacking
Prucalopride	5-HT_4_ receptor agonist	2 mg (tablet)	Currently under investigation for diabetic gastroparesis in phase III trials. May be used off-label in selected cases
Granisetron	5-HT_3_ receptor agonist	3.1 mg per 24 hours (patch)	Evidence from controlled trials is lacking in diabetic gastroparesis
